# Supplementary material for: Tamarind Seed Polysaccharide Hydrolysate Ameliorates Dextran Sulfate Sodium-Induced Ulcerative Colitis via Regulating the Gut Microbiota
Source: Pharmaceuticals (Basel). 2023 Aug 10;16(8):1133. doi: 10.3390/ph16081133 (PMC10459238; doi:10.3390/ph16081133)
Supplement: Supplementary file 1 [file pharmaceuticals-16-01133-s001.zip › Supplementary Materials.pdf]

Tamarind seed polysaccharide hydrolysate ameliorates dextran sulfate sodium-induced ulcerative colitis via regulating the gut microbiota

Kangjia Jiang [1], Duo Wang [1], Le Su [1], Xinli Liu [1], Qiulin Yue [1], Baojun Li [2], Kunlun Li [2], Song Zhang [1,*] and Lin Zhao [1,2,*]

1 State Key Laboratory of Biobased Material and Green Papermaking, School of Bioengineering, Qilu Uni-versity of Technology, Shandong Academy of Sciences, Jinan, 250353, China; jiangkangjia0813@163.com (K.J.); 1015348528@qq.com (D.W.); sule@sdu.edu.cn (L.S.); vip.lxl@163.com (X.L.); yueqiulin88@163.com (Q.Y.); 13869149205@163.com (B.L.); li_kunlun@163.com (K.L.).

2 Shandong Chenzhang Biotechnology Co., Ltd., Jinan, 250353, China

* Correspondence: zhangsrz@163.com; Tel.: +86-18653114018 (S.Z.); iahb205@163.com; Tel.: +86-13953107589 (L.Z.)

Supplementary Materials:

Table S1. Sequences of Mouse Primers used for RT-PCR Analysis

Gene	Forward primer (5′-3′)	Reverse primer (5′-3′)
GADPH (internal referenc e gene)	TGTGTCCGTCGTGGATCTGA	TTGCTGTTGAAGTCGCAGGAG
ZO-1	GCGAACAGAAGGAGCGAGAAGA G	GCTTTGCGGGCTGACTGGAG
Occludi n	TGGCTATGGAGGCGGCTATGG	AAGGAAGCGATGAAGCAGAAGG C

Table S2. *PCR amplification system*

Components	Dose
SYBR (2 ×)	10 μL
PCR Forward Primer (10 μM)	0.4 μL
PCR Reverse Primer (10 μM)	0.4 μL
Sterile Water	7.2 μL
cDNA	2 μL

Table S3. *PCR amplification program*

Reaction stage	Temperature	Time	Number of Cycles
Initial Denaturation	95°C	5 min	1
Denaturation	95°C	30 s	40

Primer Annealing	58°C	30 s	
Extension	72°C	30 s	
Extension	72°C	5 min	1

Supplementary Materials and Methods:

4.5. HPLC-ESI/MS Analysis

The method of HPLC-ESI/MS analysis was carried out on a liquid chromatograph-mass spectrometer (Waters Alliance, Waters Corporation, USA) equipped with a RP C18 column (5.0 μm, 250 mm × 4.6 mm inner diameter (i.d.),Agilent). The experimental conditions were as follows: mass scanning range m/z 200-2000; N2 as the sheath gas (flow rate: 30 arb) and auxiliary gas (flow rate: 5 arb); capillary temperature and voltage of 360 °C and 40 V, respectively; injection volume: 25 μL

4.11 Determination of SCFAs

Colon contents (100 mg) were mixed with 0.5 mL of distilled water in a 2 mL Eppendorf tube, and the mixture was mixed by vortexing for 5 min, followed by centrifugation at 3,000g for 15 min at 4°C. The obtained supernatant (200 μL) was mixed with 20 μL of 2-ethylbutyric acid (internal standard), 0.5 mL hydrochloric acid (37%), and 2 mL diethyl ether by vortexing for 3 min. After centrifugation, anhydrous sodium sulfate (Na2SO4) was added to the resulting supernatant and vortexed for 3 min. After standing for 10 min, the supernatant was purified by filtration through an organic membrane of 0.22 micron. Typical operating conditions were as follows: The column temperature was set at 100°C for 30 s, then increased to 200°C at 8°C/min with a hold time of 2 min, and finally increased to 240°C at 10°C/min with a hold time of 1 min. The electron ionization energy was set at 70 eV.

4.12 High-throughput sequencing of Gut Microbiota

341F (5′-CCTAYGGGRBGCASCAG-3′) and 806R (5′-GGACTACNNGGGTATCTAAT-3′) primers were used to PCR amplify the V3-V4 variable region. The PCR products were mixed at equal concentrations according to the PCR product concentrations, and then purified by agarose gel electrophoresis using 1 × TAE at 2% concentration, and the target bands were selected for recovery from the cut gels. The product purification kit used was Qiagen's Qiagen Gum Recovery Kit (Qiagen, Germany). Library construction was performed using Illumina TruSeq® DNA PCR-Free Sample Preparation Kit (Illumina, San Diego, USA). The constructed libraries were quantified and library tested by Qubit (Thermo Scientific), and then sequenced using NovaSeq 6000 PE250. Sequencing service and data analysis service were provided by Wekemo Bioincloud (Shenzhen China)
