# Supplementary material for: Tamarind Seed Polysaccharide Hydrolysate Ameliorates Dextran Sulfate Sodium-Induced Ulcerative Colitis via Regulating the Gut Microbiota
Source: Pharmaceuticals (Basel). 2023 Aug 10;16(8):1133. doi: 10.3390/ph16081133 (PMC10459238; doi:10.3390/ph16081133)
Supplement: Supplementary file 1 [file pharmaceuticals-16-01133-s001.zip › WB.pdf]

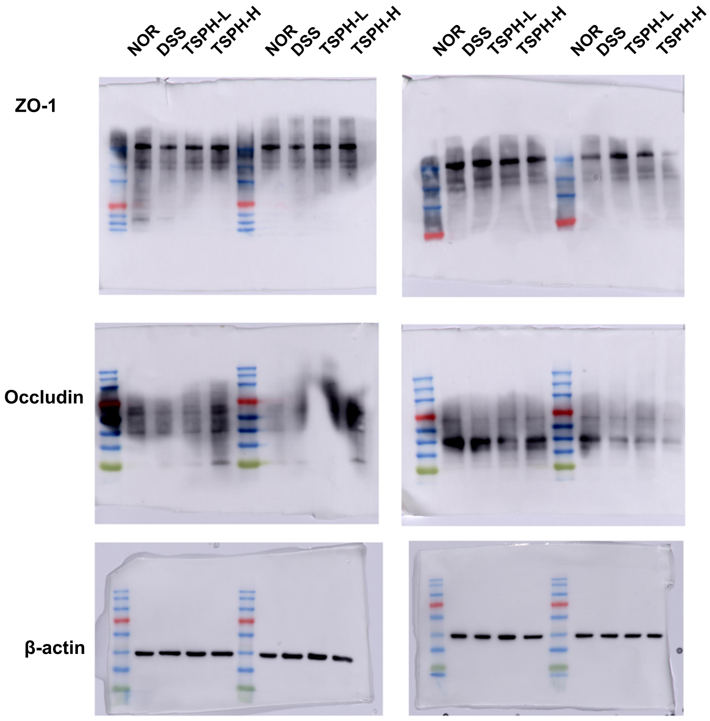

corresponding to the

Figure5 in the main article
